# Common assumptions in tobacco control that may not hold true for South-East Asia

**DOI:** 10.1016/j.lansea.2022.100088

**Published:** 2022-10-12

**Authors:** Kamran Siddiqi, Monika Arora, Prakash C. Gupta

**Affiliations:** a Department of Health Sciences, University of York and Hull York Medical School, Seebohm Rowntree building, University of York, York YO10 5DD, United Kingdom; b HRIDAY, New Delhi, and Public Health Foundation of India, New Delhi, India; c Healis Sekhsaria Institute for Public Health, Thane, Maharashtra, India

## Abstract

Tobacco is a threat to public health in South-East Asia and its control should be a priority. However, many common assumptions about tobacco control may not hold true for the region and can misdirect policy. The substantial health risks associated with smokeless tobacco have been largely misunderstood and neglected. The syndemic association between tuberculosis and tobacco has also been overlooked. Similarly, less attention has been paid to address second-hand smoke exposure of pregnant women to indoor smoking (caused predominantly by men). On the other hand, our poor understanding of the diverse tobacco supply chain has been blocking progress in tobacco control. Finally, the rising popularity of electronic cigarettes has thrown new challenges; many governments, concerned for its youth, have banned such products. We argue for a nuanced approach to tobacco control in South-East Asia. We also encourage a wider debate in public health, where other established assumptions may be hampering progress.

*“All generalisations are dangerous, even this one”*

Alexandre Dumas

Tobacco use is devastating public health and the economy across the globe and the WHO South-East Asia Region (SEAR) is at the epicentre of this threat.^1^ Out of 1·3 billion tobacco users, >400 million reside within the 11 SEAR countries (Bangladesh, Bhutan, North Korea, India, Indonesia, Maldives, Myanmar, Nepal, Sri Lanka, Thailand and Timor-Leste).^1^ In 2019, out of 8 million tobacco-related deaths, >2 million occurred in this region.^2^ Compared to other WHO regions, SEAR reported the highest prevalence of tobacco use (27·9%; males 46% and females 9·7%).^1^ The region is also a hub for tobacco growers and manufacturers with India and Indonesia being among the top five tobacco producing countries in the world.^3^

The ratification of the WHO Framework Convention on Tobacco Control (FCTC) by all SEAR countries except Indonesia, makes tobacco control a priority by the respective governments. Yet progress is slow, and many tobacco control policies are not well-developed in most countries. The 2021 WHO report on the global tobacco epidemic highlights a significant gap between definition and enforcement of tobacco control policies.^1^ Among factors responsible for this lack of progress, certain key assumptions about tobacco use and its control might be at play. Cited commonly in the literature, these assumptions may not hold true for the region yet remain entrenched in tobacco-related research and policy paradigms. From a SEAR perspective, these may be inaccurate and could mislead effective policy formulation and its implementation. In this paper, we highlight a few common assumptions, comment on their origin, demonstrate why these assumptions cannot be generalised to SEAR and show how these might mislead policy direction. By discussing a few examples, we examine tobacco control from a SEAR perspective mainly to encourage further debate in not just tobacco control but also in wider global health. We hope that by examining such issues from a regional perspective, we may be able to challenge certain generalisations in other health areas too.

## What's in a name?

We mostly speak of tobacco but use the term smoking synonymously. Smoking has long been used instead of tobacco in scientific literature, campaign materials, legislature, policies, and services. This may not be problematic for countries where smoking is the only or principal form of tobacco consumption; could even be desirable to use colloquial terms. However, in reality, this would apply to very few countries. In SEAR where the majority consume tobacco in non-combustible forms,^4^ use of the term smoking as a synonym for tobacco is reductive. With the exception of smoke-free laws, its use in tobacco control laws is particularly problematic. In a recent review, we found several countries using a narrow definition (often restricted to smoking) in their legislations hence undermining the comprehensive nature of the WHO FCTC.^5^ Beyond semantics, many countries e.g. Bangladesh, until recently, mentioned tobacco in certain legislations and smoking in others, throwing confusion for the regulators on one hand and offering escape clauses for tobacco manufacturers on the other.^6^ In Malaysia (included in the UN classification of Southeast Asia), law mandates that health warnings appear on cigarette packages only.^7^ Many other countries where people of South Asian-origin reside as minorities and consume smokeless tobacco (ST) products, legislation, campaigns and services continue to use the term smoking.^8^ In the UK, the first policy paper on tobacco was titled ‘Smoking Kills’,^9^ and cessation services are still called Stop Smoking Services. On the other hand, a Philip Morris backed foundation called itself as Smokefree World consciously indicating a shift to non-combustible tobacco products but with no intention of stopping manufacturing tobacco anytime soon.^10^ We recommend all policy makers and legislators to comply with FCTC and follow the WHO lead who has for long adopted tobacco use as the default terms and defines tobacco as “products entirely or partly made of the leaf tobacco as raw material which are manufactured to be used for smoking, sucking, chewing, or snuffing”.

## Smokeless tobacco (ST) is not a harm reduction product in SEAR

**Fig. 1:**
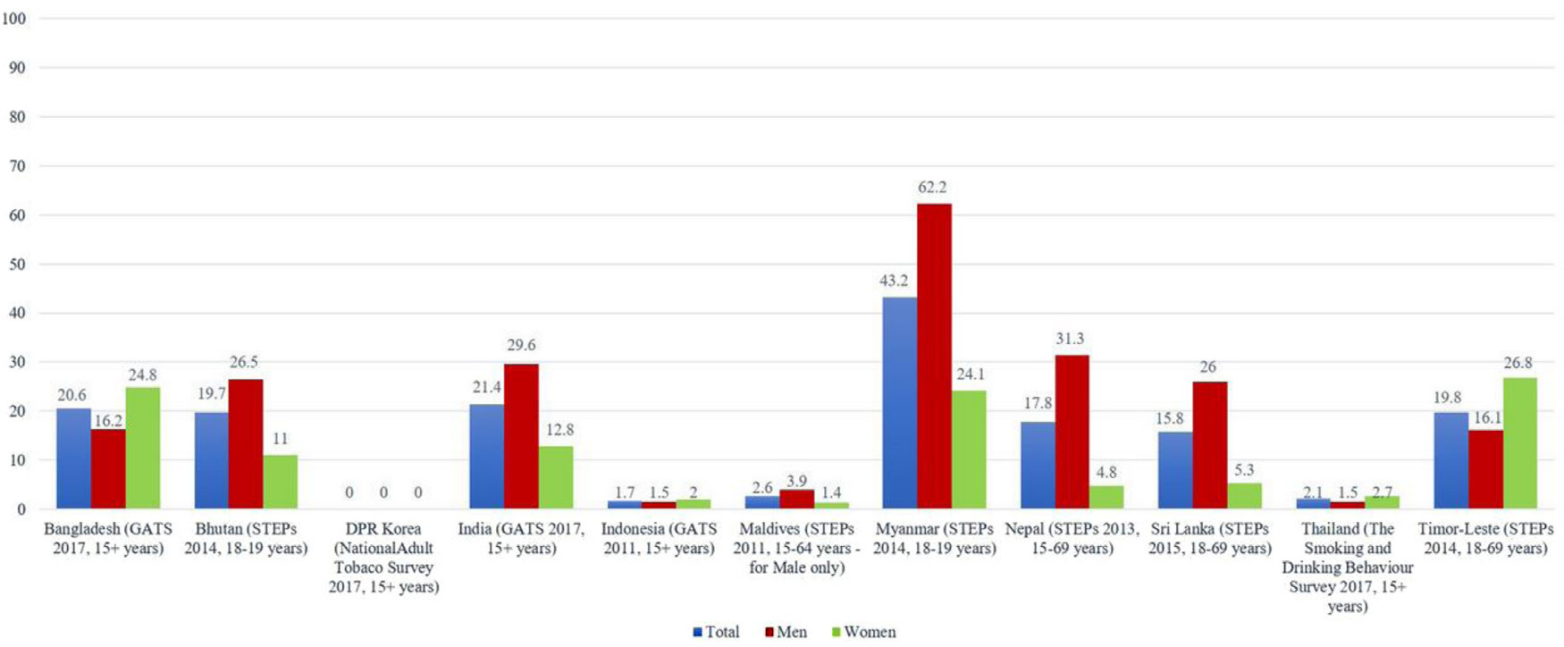
**Percentage of current ST users among adults in SEAR**.^4^

The underlying idea of harm reduction involves presenting a product with less adverse health effects as a substitute for a product with more severe negative health effects.^11^ Evidence suggests that ST releases nicotine at a slower rate than smoking, resulting in lower peak arterial nicotine levels and avoids the inhalation of combustion fumes and particulates.^11^ Hence, ST has been advocated as a cigarette substitute to reduce tobacco-related harm in Sweden, the US and few high-income countries where smoking is the most prevalent form of tobacco use.^12^^,^^13^ Products, such as Swedish snus have lower disease risks than the low-cost ST products used in SEAR.^13^ On the other hand, the relative risks and the population attributable fractions are much higher for SEAR nations, where 85% of global ST users live,^4^ with Myanmar having the highest prevalence of ST users (Fig. 1).^4^ Hence, endorsing ST products as harm reduction products in SEAR nations may undermine current tobacco control efforts and encourage ST use, where ST consumption is far more prevalent and the associated health risks are much higher than Sweden and the US.^13^ The higher disease risk is explained by the elevated levels of nitrosamines and other toxic chemicals in ST products available in SEAR and also by the less favourable socioeconomic conditions that interact with tobacco health effects and increase morbidity and mortality.

ST contributes to a significant number of deaths and DALYs worldwide, with SEAR bearing 85% of this burden.^4^^,^^14^ ST contains approximately 28 different carcinogens that result in higher cancer rates^15^ with oral cancer being the most common form of cancer, accounting for over 70,000 head and neck cancer cases each year in SEAR.^4^ Of all SEAR nations, oral cancer is the third most common form of cancer in India,^15^ with significant proportion of incident cancers of others such as pharynx, larynx, oesophagus, and stomach. A systematic review by Sinha and colleagues revealed a significant association for oral—5·55 (5·07, 6·07), pharyngeal—2·69 (2·28, 3·17), laryngeal—2·84 (2·18, 3·70), oesophageal—3·17 (2·76, 3·63) and stomach—1·26 (1·00, 1·60) cancers.^16^ Apart from cancers of the upper aerodigestive tract, ST use has also been attributed to cancers of the pancreas, uterine cervix, stomach, and cardiovascular deaths, and poor pregnancy outcomes.^16^ This evidence clarifies why ST cannot be accepted as a harm reduction product in this part of the world.

**Fig. 2:**
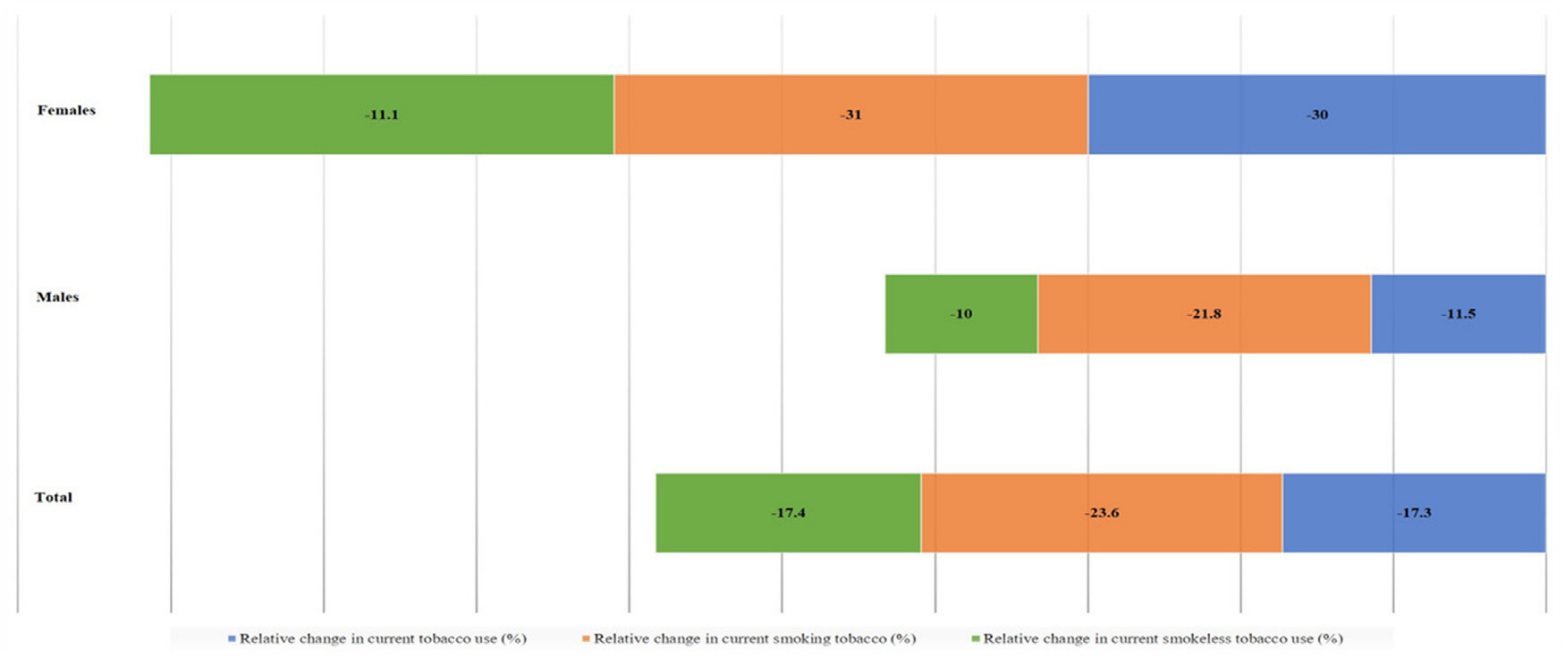
**Relative change in prevalence of different tobacco products in India among adults aged more than 15 years from 2009-10 to 2016-17**.^17^^,^^18^

**Fig. 3:**
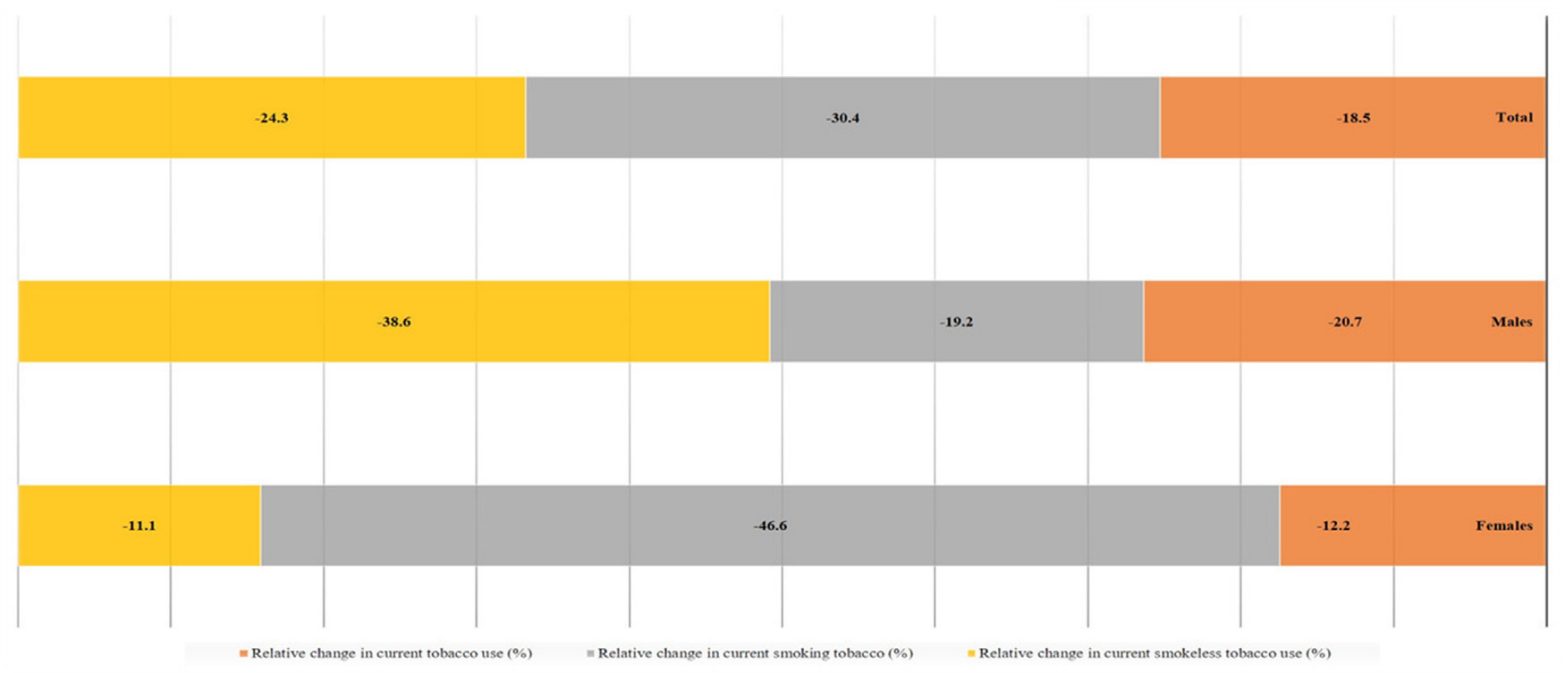
**Relative change in prevalence of different tobacco products in Bangladesh among adults aged more than 15 years from 2009 to 2017**.^19^^,^^20^

Over the years, prevalence of tobacco use across products has reduced; but the relative change for smoking is more significant as compared to ST, as reported among adults aged more than 15 years in India,^17^^,^^18^ and Bangladesh,^19^^,^^20^ (Fig. 2, Fig. 3). This differential shift has a gender dimension too; for example, in Bangladesh (where more women [24·8%] than men [16·8%] use ST) a slower decline in ST use in general as well as compared to men (Fig. 3), would enhance health disparities. This underscores the need to address ST according to context under comprehensive tobacco control strategy.

Among all SEAR countries, India has made the most progress in ST control with eighteen Indian states banning gutka under the new Food Safety and Standards Authority of India (Prohibition and Restrictions on sales) Regulations (FSSAI), 2011. The section 2·3·4 under this regulation prohibits the use of tobacco and nicotine as ingredients in any food products and thus sale of all food products containing tobacco, such as gutka and pan masala.^21^ Other SEAR nations like Nepal have prohibited the use of all tobacco products in public places including ST. However, the ST products are largely unregulated as there is limited evidence on the properties, production, and its ingredients.^5^

## Health consequences of tobacco – what is missing?

Tobacco use leads to terrible consequences mediated primarily via tobacco-induced diseases.^22^ The health impact is often represented in terms of tobacco-related disease burden, which in turn is estimated as the attributable fraction of mortality and morbidity associated with tobacco-induced diseases.^22^ Until recently, major reports on the health consequences of tobacco,^23^ were based primarily on studies from high-income countries.^24^^,^^25^ These reports rarely highlighted those tobacco-induced conditions, which were relatively uncommon in high-income countries as compared to other regions e.g. SEAR. For example, tuberculosis (TB) was completely missed among the list of tobacco-induced diseases in many highly cited reports,^23^ public information resources,^26^ and manuscripts.^27^ On the other hand, conditions such as chronic obstructive pulmonary disease have been cited as common tobacco-induced disease without qualifications. In fact, TB accounts for nearly a third of all tobacco-related deaths in India,^28^ and this is in equal proportions to tobacco-related deaths caused by cardiovascular diseases. Furthermore, it is commonly assumed that lung cancer is the most common cancer caused by tobacco use, which is correct for countries where smoking is the predominant form of tobacco use.^23^ However in countries in Southeast Asia (as per United Nations[UN] classification), oral cancer is the leading cause of all cancer-related deaths among males and is the most common tobacco-related cancer in both sexes.^29^ This is mainly due to the fact that ST is the predominant form of tobacco use in SEAR and oral cancer is its most common serious consequence.^16^^,^^30^

If key tobacco-induced diseases are missed or rarely mentioned in the literature, then this has serious consequences for regional policy and practice. First, one cannot estimate an accurate impact of tobacco use in SEAR without taking account of all relevant tobacco-induced diseases and understanding their relative and absolute risks. Second, any regional disease burden estimates attributed to tobacco must include all forms of tobacco including ST. In a recent estimate, the ST disease burden in SEAR was found considerable and on the rise.^4^ Third, due to lack of awareness, disease control programmes such as TB largely ignore the role played by smoking or any need to help stop its use among TB patients and those suspected.^31^ In recent years, there has been an increasing awareness of the role of tobacco use in inducing TB,^32^ the opportunities for TB health professionals to support tobacco cessation,^31^ and the improvements observed in TB outcomes as a result of stopping smoking.^33^ The emphasis and investment in TB control in SEAR can be further guided to address tobacco use as a key intervention to control TB.

While not induced by tobacco, its use leads to terrible consequences for people living with HIV and AIDS.^34^ With India being home to over 2 million people living with HIV and AIDS,^35^ tobacco control can play a significant and useful role in HIV and AIDS control.^36^ With a sharper focus on regional context, more effective policy direction can be found with the alignment of tobacco control with TB and HIV programmes.

The use of ST may also increase the spread of communicable diseases. Evidence shows that ST consumption involves placing these products inside the mouth over a long period of time that induces salivation and hence increased spitting in public areas, owing to the possibility of spreading communicable diseases such as SARS-CoV-2 virus and TB.^37^, ^38^, ^39^ In the view of COVID-19 pandemic, India prohibited the sale of tobacco products during the COVID-19 lockdown to prevent the spread of the SARS-CoV-2 virus.^37^ In addition to the added disease burden due to high prevalence of certain conditions (TB, HIV) in SEAR, tobacco death toll and morbidity may be higher in the region due to social deprivation and less favourable social determinants of health.

## Second-hand smoke exposure - smoke-free laws and beyond

Smoking bans in workplaces, bars and restaurants were first introduced in Ireland in 2004.^40^ In quick succession, most high-income countries introduced comprehensive smoke-free laws resulting in significant reductions in second-hand smoke (SHS) exposure,^41^ and improvements in health outcomes,^42^ especially among children.^43^ It was assumed that if the rest of the world followed suit, the health impact would be transformational. Nearly two decades on, more than three-quarters of the world population remains unprotected from SHS exposure.^1^ An absence and/or poor enforcement of comprehensive smoke-free laws are commonly quoted as the reasons for this failure.^44^ The situation in SEAR is no better; while smoking is banned in health and educational facilities in the entire region, many countries are yet to implement smoking bans in indoor workplaces, restaurants, bars and other entertainment facilities.^1^ In India, where smoking bans are fairly comprehensive, 30-40% population remains exposed to SHS in restaurants and workplaces.^45^ In Bangladesh where smoking is banned in educational institutions, 95% primary schoolchildren in Dhaka were found cotinine positive highlighting recent exposure to SHS.^46^ Moreover, a synergistic interaction between SHS and high levels of ambient air pollution observed in cities and indoor air pollution due to biomass fuel in Bangladesh,^47^^,^^48^ and other SEAR countries is likely to enhance the associated health risks particularly chronic respiratory and heart conditions. Legislating to implement comprehensive smoke-free laws remains a key action to protect against SHS but on its own, this may not be sufficient to protect non-smoking populations from smoking-related harms in SEAR. In the absence of a desire or resources to police smoking bans across this vast region, more evidence-based approaches are needed to empower communities and families to expect smoke-free environments in public as well as private spaces.

SHS exposure and smoke-free laws accentuate gender inequity in LMICs -most evident in SEAR. While smoking is predominantly a male behaviour -30·5% men smoke as compared to 1·5% women in SEAR^49^- females are more likely to be exposed to SHS at homes than males.^45^ In SEAR, 57% women reported exposure to SHS on a daily basis while pregnant almost always due to men smoking in their homes.^50^ As a result of such high exposure levels during pregnancy, a significant proportion of stillbirths in SEAR (ranging from 14% in Indonesia to 7% in Bangladesh) may be due to SHS.^50^ Given that the smoke-free laws do not extend to private homes where more women are exposed to SHS, they are left with no policies to protect them and their newborn babies from the related harms. The cultural contexts in SEAR have served to inhibit smoking among women, but their limited social, political and economic power, has reduced their ability to challenge prevalent male smoking behaviours and protect themselves and their newborn babies from SHS.^51^ A narrow focus on high income countries has resulted in neglecting this issue in policy arena and further efforts are needed to develop evidence and approaches to reduce women's exposure in household.

## Tobacco uptake in youth - what may or may not work?

The rise of ST consumption alongside smoking among adolescents aged 13-15 years in some SEAR countries is a cause of concern. Countries like India have also reported ST use initiation at age as young as 10 years.^52^ The prevalence of current use of ST among adolescents varies from 2·7% in Thailand to 46·1% in Maldives. Among boys, ST prevalence ranges from 4·1% in Thailand to 47·5% in Maldives and among girls, from 1·3% in Thailand to 44·6% in Maldives.^53^ In Timor-Leste, cigarettes are the predominant form of tobacco use; in a 2013 survey of 13–15-year-old students 42.4% reported tobacco use and 28.9% cigarette smoking.^54^ Tobacco control interventions have shown reduction in tobacco use among adolescents but the reduction has been higher for smoking forms as compared to ST use. A cluster randomised controlled-trial ACTIVITY among adolescents in a community setting, conducted in Delhi, India revealed no differences in ST use among for both intervention and control group.^55^ Likewise, project MYTRI was equally unsuccessful in lowering ST usage among adolescents in the study schools of Delhi and Chennai, India, whereas significant reduction was reported for smoking tobacco forms.^56^ Despite a comprehensive tobacco ban, ST use among adolescents in Bhutan went up (from 14.5% to 25.0% in boys and from 6% to 18.9% in girls) between 2006 and 2013.^57^ Thus highlighting the need for more intensive interventions, like tax increase, comprehensive advertising prohibitions, large media campaigns and other population level policy interventions to address tobacco use in this context.

## Tobacco industry - big tobacco and beyond

Among SEAR nations, India is the second largest producer and consumer of tobacco in the world,^58^ where ST industry is highly fragmented as some products are commercially manufactured and rest made under cottage industry and sold locally.^59^^,^^60^ ST industry uses several tactics to recruit customers including: event sponsorships, colourful display of product packages in strings at point of sale and misleading taglines in advertisements and promotions.^21^ SEAR nations like India, Maldives, Nepal and Sri Lanka have made substantial strides with the introduction of 80-90% of pictorial health warnings on ST products.^61^ India ratified the WHO FCTC and enacted Cigarettes and Other Tobacco Products Act (COTPA) 2003, which prohibits tobacco product advertising both directly and indirectly. However, the ST industry has continued to flout advertising bans through brand stretching, using same brand names for tobacco and non-tobacco products.^60^ Despite the fact that gutka brands are not advertised, identical brands of pan masala sans tobacco are advertised.^21^^,^^60^ Moreover, the ST industry uses small packaging that makes the product easily accessible, affordable and reduces the impact of graphic health warnings. The Trademarks Act also permits the ST industry to evade the law and advertise the same brand for non-tobacco items.^21^ Though tobacco use in dentifrices is prohibited by law, they are available as tobacco paste brand.^60^^,^^62^ In short, the diversity observed among tobacco products in SEAR also extends to the tobacco industry ranging from Big Tobacco to small unlicensed manufacturing units. As we learn to counter industry tactics and interference in policy, we must also take a closer look at the dynamics of the ST and bidi cottage industry and find potential regulatory solutions that could curb the use of all forms of tobacco.

## Illicit tobacco trade - beyond cigarette prices

Illicit tobacco trade makes cheap cigarettes more accessible and hence promotes their consumption.^63^ Many high income countries e.g., Italy, Spain and the UK, have been successful in curbing illicit tobacco trade.^63^ Based on such experiences, the WHO launched a Protocol to Eliminate Illicit Trade in Tobacco Products in 2013 -a legal instrument to promote international and intersectoral cooperation to address this global iusse.^64^ Most SEAR countries lack the capacity to curb illicit tobacco trade,^65^ and so far only two (India and Sri Lanka) have signed the Protocol. Furthermore, certain assumptions made about the drivers, magnitude, and policies to address illicit tobacco trade may not be accurate or feasible for SEAR. For example, lucrative profit margins by selling cheap cigarettes in countries with high cigarette prices is considered as the key driver for illicit tobacco trade.^63^ Tobacco industry has often used this argument, as a tactic to prevent governments from increasing tobacco taxes and prices.^66^ This may not be the case for SEAR where illicit tobacco trade is rife but cigarettes are relatively cheap, tobacco taxes are low and the tobacco market is dominated by bidi and ST, which are even cheaper. Other factors including social acceptance of illicit trade, weak governance, regulatory frameworks and tax administration and the proliferation of diverse informal manufacturers and distribution networks could be more important determinants of illicit tobacco trade in SEAR.^67^ ST and bidi -manufactured primarily in informal sector- are cheap and taxed well below cigarettes. A lack of compliance of its packaging and labelling features with in-country legislation indicates that ST supply chain is largely unregulated.^68^ It is no surprise that no government in SEAR has neither been able to estimate the magnitude of illicit trade of all forms of tobacco nor made any in-roads to curb it.

We recommend that all SEAR countries ratify the Protocol to Eliminate Illicit Trade in Tobacco Products, however, this may not be sufficient on its own. Strengthening tax administration is a crucial step in eliminating illicit tobacco trade, the feasibility of which remains questionable in relation to bidi and ST. Most SEAR countries will also need to modify their legal, administrative and enforcement structures,^69^ which may include formalising the bidi and ST supply chain including issuing and enforcing vendor licences.

## Electronic cigarettes

The emergence of electronic cigarettes and other novel nicotine and tobacco products has challenged the global consensus on tobacco control efforts.^70^ While there is a general agreement on the risks of e-cigarette uptake among youth,^71^ opinions on its usefulness in helping people to quit smoking are split.^72^ In the absence of generalisable evidence and a lack of global consensus, countries have adopted a range of policies stretching from a complete ban to promoting electronic cigarettes for smoking cessation.^73^ A conceptual dichotomy has emerged; those considering electronic cigarettes as tobacco products are borrowing policies from tobacco control which may or may not be appropriate. Similarly, those approaching them as nicotine delivery systems only have devised specific regulations, which are novel, and the supporting evidence is limited and contextual. In the meantime, the tobacco industry has rapidly taken over e-cigarettes market and are publicising it in many ways including as harm reduction products.

Electronic cigarettes are also gaining popularity in the region; for example, Malaysia (included in the UN classification of Southeast Asia) is rapidly becoming a lucrative market for electronic cigarettes.^74^ This growth poses its own specific challenges for policy makers and it will be difficult to transfer evidence emerging from high income countries. Most SEAR countries have high tobacco-burden, and some are still in the early stages of the tobacco epidemic. As shown above, most have not fully implemented WHO MPOWER strategies e.g., tobacco taxation. The rise of electronic cigarettes in this epidemiological and political backdrop may lead to multiple effects ranging from public confusion over health risks to diverted policy and regulatory focus. Many SEAR countries, with limited capacity to implement and monitor tobacco control regulations and having seen the impact of ST products, are legitimately concerned about regulating the rapidly emerging market of novel nicotine and tobacco products. It is with this concern, most SEAR countries (Bhutan, India, Nepal, Sri Lanka, Thailand, Timor-Leste) have banned electronic cigarettes completely. Even though bans are in place e-cigarettes are still available through online platforms. Bans on tobacco products have the tendency to backfire too as in the case of Bhutan where despite a total ban on selling tobacco, its use increased.^75^ Illicit sales may flourish under a ban that is not complied with and populations may continue to be exposed to cheap unregulated products without any support for quitting. While such violations need to be addressed through rigorous monitoring and enforcement mechanisms, SEAR countries may have to find further solutions to regulate e-cigarettes and other emerging novel nicotine products.

## Conclusions

Tobacco control in SEAR countries is complex due to the myriad forms in which tobacco is manufactured, marketed, and sold. These products, their use and exposure patterns have emerged within SEAR countries’ specific demographic, historical and socio-cultural context. The endemic nature of certain communicable diseases such as TB poses tobacco-related threats that have not been thought of and hence neglected in the past. This unique scenario requires contextualised solutions to be designed and implemented from WHO FCTC and beyond. Monitoring usage and tracking ST products is an immediate priority with countries giving due attention to tackle ST alongside smoking products. ‘Outside the box’ solutions such as banning spitting in public use to control ST use- related infections may be employed under the Anti-Spitting Act and not Tobacco Control Act. Similarly, e-cigarettes are banned under a separate Act, thus requiring going beyond WHO FCTC to comprehensively design policies to the SEAR context.

## Contributors

KS conceived the idea and conceptualised, drafted, edited, and approved the manuscript. MA drafted, edited, and approved the manuscript. In addition, MA analysed the data to produce Fig. 1, Fig. 2, Fig. 3. PCG identified useful literature, reviewed, edited, and commented on the draft and approved the final version.

## Data sharing statement

The data used in the manuscript and the Fig. 1, Fig. 2, Fig. 3 are based on publicly available sources accessible through citations appearing in the manuscript.

## Declaration of interests

KS and MA - time for this research was funded by the National Institute for Health Research (NIHR) [ASTRA (Grant Reference Number 17/63/76)] using UK aid from the UK Government to support global health research. The views expressed in this publication are those of the author(s) and not necessarily those of the NIHR or the UK Department of Health and Social Care. All authors do not have any other conflict of interests.
